# Human Leucocyte Antigen G and Murine Qa-2 Are Critical for Myeloid Derived Suppressor Cell Expansion and Activation and for Successful Pregnancy Outcome

**DOI:** 10.3389/fimmu.2021.787468

**Published:** 2022-01-17

**Authors:** Stefanie Dietz, Julian Schwarz, Ana Velic, Irene González-Menéndez, Leticia Quintanilla-Martinez, Nicolas Casadei, Alexander Marmé, Christian F. Poets, Christian Gille, Natascha Köstlin-Gille

**Affiliations:** ^1^ Department of Neonatology, Tuebingen University Children’s Hospital, Tuebingen, Germany; ^2^ Interfaculty Institute for Cell Biology, Proteome Center Tuebingen (PCT), University of Tuebingen, Tübingen, Germany; ^3^ Department of Pathology, University of Tuebingen, Tübingen, Germany; ^4^ Next Generation Sequencing (NGS) Competence Center Tuebingen (NCCT), Tuebingen, Germany; Institute of Medical Genetics and Applied Genomics, University of Tuebingen, Tuebingen, Germany; ^5^ Gynecology and Obstetrics Practice, Am Lustnauer Tor, Tuebingen, Germany; ^6^ Department of Neonatology, Heidelberg University Children’s Hospital, Heidelberg, Germany

**Keywords:** HLA-G, Qa-2, pregnancy, myeloid-derived suppressor cells, abortion, preeclampsia

## Abstract

During pregnancy, maternal immune system has to balance tightly between protection against pathogens and tolerance towards a semi-allogeneic organism. Dysfunction of this immune adaptation can lead to severe complications such as pregnancy loss, preeclampsia or fetal growth restriction. In the present study we analyzed the impact of the murine MHC class Ib molecule Qa-2 on pregnancy outcome *in vivo*. We demonstrate that lack of Qa-2 led to intrauterine growth restriction and increased abortion rates especially in late pregnancy accompanied by a disturbed trophoblast invasion and altered spiral artery remodeling as well as protein aggregation in trophoblast cells indicating a preeclampsia-like phenotype. Furthermore, lack of Qa-2 caused imbalanced immunological adaptation to pregnancy with altered immune cell and especially T-cell homeostasis, reduced T_reg_ numbers and decreased accumulation and functional activation of myeloid-derived suppressor cells. Lastly, we show that application of sHLA-G reduced abortion rates in Qa-2 deficient mice by inducing MDSC. Our results highlight the importance of an interaction between HLA-G and MDSC for pregnancy success and the therapeutic potential of HLA-G for treatment of immunological pregnancy complications.

## Introduction

Premature termination of pregnancy either by abortion or by preterm delivery is the most important pregnancy complication. About 15% of clinically recognized pregnancies miscarry, however total reproductive losses are closer to 50% (1). Preterm birth, defined as birth before 37 weeks of gestation, affects 5% to 18% of successful pregnancies (2). Besides chromosomal or anatomic anomalies and endocrinological disorders, immunological factors play an important role in pathogenesis of abortions and preterm delivery (2). During pregnancy, there is a close contact between maternal immune cells and fetal cells. Thus, the maternal immune system has to balance tightly between protection against pathogens and tolerance towards the semi-allogeneic fetus. Dysfunction of the immune adaptation to pregnancy can lead to severe complications such as pregnancy loss, preeclampsia, preterm birth or fetal growth restriction. The mechanisms facilitating maternal-fetal tolerance are only incompletely understood and therapeutic options are limited.

The major histocompatibility class Ib (MHC Ib) molecule human leucocyte antigen G (HLA-G) is a non-classical MHC I molecule with low allelic variation and a restricted peptide repertoire which was first described by Geraghty et al. in 1987 (3, 4). Under physiological conditions, HLA-G is mainly expressed by trophoblast cells at the maternal-fetal interface (5) and can be secreted in a soluble form (soluble HLA-G, sHLAG) to the circulation (4). *In vitro* studies show that HLA-G shapes the maternal immune system towards tolerance by modulating function of antigen-presenting cells (APCs) (6) and inhibition of T-cell activity (7) and natural killer cell (NK-cell) cytotoxicity (8). During an uncomplicated pregnancy, levels of sHLA-G first increase and then decrease until the third trimester. Undetectable sHLA-G levels or variation in the course of sHLA-G levels seem to be related with gestational complications such as pregnancy loss and preeclampsia (9, 10). However, so far there have been no *in-vivo* studies that clearly demonstrate a beneficial role of HLA-G for pregnancy success.

Qa-2 is a murine non-classical MHC-Ib molecule which controls the rate of preimplantation embryonic cleavage division and subsequent embryo survival. It is the product of the preimplantation embryo development (Ped) gene (11) and encoded by four genes, Q6, Q7, Q8 and Q9, of which only Q7 and Q9 are transcribed in mouse embryos (12, 13). Embryos expressing Q7 or Q9 or both show the *Ped fast* phenotype with high embryo cleavage rates, while mouse strains that have a deletion of both genes do not express Qa-2 protein and show the *Ped slow* phenotype with slowed preimplantation embryo development and survival (12, 14). Due to various similarities between human HLA-G and murine Qa-2, such as their occurrence as membrane-bound and soluble forms and profound immunoregulatory properties of both molecules, Qa-2 has been considered a possible candidate for a murine HLA-G correlate (11, 15–17). Functional characteristics of HLA-G and Qa-2 are very similar during pregnancy; both HLA-G and Qa-2 are expressed by preimplantation embryos and seem to play an important role for early embryo survival and fetal growth (18–21). However interestingly, also women with deletion of HLA-G and mice completely lacking Qa-2 can give birth to healthy offspring, suggesting that HLA-G and Qa-2 are not necessary for reproductive success but reduce it when absent (13).

The pioneer work by Carol Warner and colleagues originally characterized the Ped gene and its product Qa-2 more than 30 years ago (22, 23) and already showed that mice not expressing the Qa-2 antigen (Qa-2^-^) have smaller litters, lighter fetuses and a shorter duration of gestation than Qa-2 expressing animals (20, 24). Furthermore, they showed that Qa-2^-^ animals develop increased systolic blood pressure when they get older (25). However, until now, mechanisms mediating the protective role of Qa-2 during reproduction and especially the impact of Qa-2 on immune regulation during pregnancy are marginally understood.

Myeloid derived suppressor cells (MDSC) are myeloid cells with various immune suppressive properties. They mainly consist of two subtypes named granulocytic MDSC (GR-MDSC) with phenotypic characteristics of neutrophils and monocytic MDSC (MO-MDSC) with phenotypic similarities to monocytes. In mice, GR-MDSC are defined as CD11b^+^/Ly6G^+^/Ly6Clo and MO-MDSC as CD11b^+^/Ly6G-/Ly6Chi cells (26). Primarily, MDSC accumulation has been described under tumor conditions where they suppress immune responses against tumor cells, thereby leading to disease progression (27). Later, MDSC have been shown to accumulate under various other pathologies like infection, trauma, autoimmune disease, obesity and transplantation (28). In recent years, however, there is increasing evidence that MDSC also play a physiological role during pregnancy by modulating maternal immune responses and protecting the fetus from rejection (29–33). Accumulation and activation of MDSC are driven by various factors (26). Recently, we demonstrated that HLA-G induced and activated GR-MDSC *in vitro* (34). Furthermore, it could be shown by us and others that the transcription factor hypoxia-inducible factor 1α (HIF-1α) plays a role for MDSC function during pregnancy and cancer (31, 35).

In the present study, we investigated the *in-vivo* role of HLA-G and Qa-2 for pregnancy success. Most inbred strains of mice, such as C57BL/6, carry all four genes encoding for Qa-2 (Q6/Q7/Q8/Q9) on each allelic chromosome, making a total of eight Qa-2 encoding genes and thus express the Qa-2 antigen widely in adult and embryonic tissues (12, 36). But there are also a few strains that are missing all eight genes, defining a null allele leading to lack of Qa-2 (13). We used such a murine model lacking the Qa-2 antigen (Qa-2 negative, Qa2^-^, B6.K1) (37) and could show that Qa-2 deficiency led to intrauterine growth restriction and increased abortion rates with profound changes in uterine spiral artery remodeling and trophoblast invasion as well as trophoblast morphology, indicating a preeclampsia-like phenotype. Immunological adaptation to pregnancy was imbalanced with altered immune cell and especially T-cell homeostasis and reduced T_reg_ numbers in Qa-2^-^ mice. Most impressively, there was a lack of MDSC accumulation and reduced functional activation of MDSC. We further showed that expression of Qa-2 on MDSC was regulated by the pregnancy hormone estrogen *via* the transcription factor HIF-1α. By application of sHLA-G to Qa-2^-^ mice we could reduce their increased abortion rate, an effect that was abrogated by simultaneous MDSC-depletion. Conversely, adoptive transfer of *in vitro* generated MDSC also resulted in a reduced abortion rate in Qa-2^-^ animals. Our results highlight the therapeutic potential of HLA-G for treatment of immunological pregnancy complications.

## Methods

### Study Approval

All experiments were approved by the ethics committee of Tuebingen University (682/2016BO1) for human studies or the regional council Tuebingen (K05/19M, K09/19M, K09/18G, K02/19G, K05/20G and K08/20G) for animal studies.

### Mice

B6.K1 (B6.Cg-H2^b3^/FlaCmwJ, Qa-2^-^), HIF-1α^flox^ (B6.129-Hif1a^tm3Rsjo^/J) mice and LysMcre (B6.129P2-Lyz2^tm1(cre)Ifo^/J) mice were obtained from The Jackson Laboratory (Bar Harbour, Maine, USA). C57BL/6J (WT) mice, CBA/J and DBA/2J mice were obtained from Charles River (Sulzfeld, Germany). HIF-1α^flox^ mice and LysMcre mice were crossed to get animals with deletion of HIF-1α in myeloid cells (HIF-1α^flox^/LysMcre, HIF-KO). The B6.K1 or Qa-2- mouse strain was developed in the 1976 by Dr. Lorraine Flaherty. It is originated spontaneously through intercross and backcross of the congenic strains B6.A-H2-T18^a^ (B6-Tla^a^) and B6.AK-H2^k^ (37). All animals were maintained under pathogen-free conditions in the research animal facility of Tuebingen University, Tuebingen, Germany. All experimental animal procedures were conducted according to German federal and state regulations.

Syngeneic matings of Qa-2^-^, HIF-KO and WT mice and allogeneic matings of CBA/J females with DBA/2J males were set up at 8-12 weeks of age. Male and female mice were mated 1:2 in the afternoon and plug control was carried out the next morning. Gestational ages were determined by visualizing of the presence of a vaginal plug (E0.5 = embryonic day).

Lack of Qa-2 expression in Qa-2^-^ mice was verified by flow cytometric analysis of blood leucocytes (**Supplementary Figure 1A**).

Abortion rates were determined by visual inspection of fetal-placental units and defined as ratio of resorbing units to the total number of implantation sites. Resorbing units were either dark, small and necrotic or pale, small and without visible fetus inside the amniotic cavity.

Fetal weight was determined by weighing of E18.5 fetuses immediately after removal from the amniotic cavity.

### sHLA-G Application, MDSC-Depletion and Adoptive MDSC Transfer

Biotinylated HLA-G1 monomers bound to the peptide KGPPAALTL were kindly provided by Jianhong Cao, Fred Hutchinson Cancer Research Center, Seattle, USA. HLA-G1 tetramers were produced by binding to streptavidin (Biolegend, San Diego, USA).

To test the effect of sHLA-G on pregnancy outcome in Qa2^-^ mice, pregnant mice were injected intravenously at E10.5 and E14.5 with 1 µg/g body weight HLA-G1 tetramers in 100 µl PBS or with 100 µl PBS alone (control).

For depletion of MDSC, pregnant Qa-2^-^ mice were injected intravenously at E10.5 and E14.5 with 250µg/mouse Ly6G-antibody (BioXCell, Lebanon, USA) in 100µl dilution buffer (BioXCell) simultaneously with the HLA-G application.

For adoptive MDSC transfer, MDSC were *in vitro* generated from bone marrow cells. Bone marrow cells were collected from femora, tibiae and humeri of WT mice by rinsing with PBS. BM-cells were than cultivated for four days with 100ng/ml G-CSF and 2,5ng/ml GM-CSF (PeproTech, Hamburg, Germany). After four days MDSC were enriched by removing non-adherent cells and detaching adherent cells containing MDSC by Trypsin/EDTA. By this method a purity of >92% can be achieved. Pregnant Qa-2^-^ mice were injected intravenously at E10.5 and E14.5 with 4x10^6^ *in vitro* generated MDSC in 100µl PBS or with PBS alone (control).

To test the effect of sHLA-G on pregnancy outcome in abortion-prone DBA/2J-mated CBA/J mice, plug-positive mice were injected intravenously with 1 µg/g body weight sHLA-G tetramers in 100 µl PBS or with PBS alone (control) at E0.5, E3.5, E6.5 and E9.5.

### Determination of Mouse Estrous Cycle

For identification of the mouse estrous cycle stage, female C57BL/6J mice were anesthetized with 1.5% isoflurane (CP-Pharma, Burgdorf, Germany). Blood was obtained by puncture of the retroorbital vein plexus or the tail vein and vaginal swabs were collected according to an established protocol (38). A cotton tipped swab (Applimed, Châtel-Saint-Denis, Switzerland) wetted with room-temperature physiological saline was inserted vaginally, gently turned, and then removed. The procedure was repeated twice for four consecutive days with 28 days in between.

The vaginal cells were transferred to a glass slide by rolling the swab over the slide. The slide was air dried, stained with hematoxylin-eosin (HE, Merck GmbH, Darmstadt, Germany) and viewed at 10x magnification under bright field illumination. The cycle stage was determined based on the presence or absence of leucocytes, cornified epithelial cells and nucleated epithelial cells according to (38).

### Patients

The local ethics committee approved this study (682/2016BO1) and all women gave written informed consent. From August to October 2019 peripheral blood from pregnant women (aged 18-43 years, gestational age 10 – 36 weeks of gestation) was collected during routine blood sampling. Patients suffering from severe pregnancy complications (severe infection, preterm rupture of membranes, preterm labour, preeclampsia/eclampsia), chronic diseases (autoimmune diseases, malignancies, chronic infections) or receiving immune-suppressive therapy were excluded.

### Mouse Tissue Collection and Single Cell Preparations

Non-pregnant and pregnant mice at gestational age E10.5 or E18.5 were euthanized by CO2 inhalation. Blood (0.5-1 ml) was collected immediately after death by intracardial puncture and placed into EDTA-tubes. Blood plasma was collected after centrifugation of whole blood at 400 rpm. Red blood cells were removed from whole blood by ammonium chloride lysis. Spleens were removed and tissue was pushed through a 100 µm filter (Greiner bio-one, Frickenhausen, Germany) using a syringe plunger. Red blood cells of the spleen were also removed by ammonium chloride lysis and the resulting cell suspension was again passed through a 40 μm filter (Greiner bio-one, Frickenhausen, Germany). Uterine horns were removed in toto. Fetuses and the fetal part of the placenta were dissected from uteri; blood vessels were removed. Uteri were placed into PBS, cut into 1 mm pieces and pushed through a 40 µm filter. Placentas were pushed through a 100 µm filter using a syringe plunger. Red blood cells were removed by ammonium chloride lysis and the resulting cell suspension was then passed again through a 40 µm filter. All cell suspensions were then adjusted to 1-4x10^6^ cells/ml in PBS or medium.

### Cell Isolation and Culture

Human peripheral blood mononuclear cells (PBMC) were prepared from EDTA blood samples by Ficoll density gradient centrifugation (lymphocyte separation medium, Biochrom, Berlin, Germany).

To isolate GR-MDSC from murine splenocytes, cells were labeled with Gr-1 Biotin-Antibody and isolated over Streptavidin microbeads followed by a second isolation step using Ly6G Biotin-Antibody and Anti-Biotin microbeads (modified protocol of MDSC Isolation Kit mouse, Miltenyi, Bergisch-Gladbach, Germany). Purity of GR-MDSC after separation was >90%, as determined by flow cytometry.

For isolation of CD4^+^ T-cells from murine splenocytes, cells were labeled with T cell Biotin-Antibody Cocktail followed by two consecutive Anti-Biotin magnetic bead separation steps (Miltenyi) according to the manufacturer´s instructions. Purity of CD4^+^ T-cells after separation was >90%, as determined by flow cytometry.

*In vitro* generation of murine MDSC was performed according to previously established protocols (31, 39). For *in vitro* generation of MDSC, non-pregnant WT and Qa-2^-^ mice were euthanized and femora and tibia removed. Bone marrow was collected by flushing the bones with PBS using a syringe and a 25G needle. Bone marrow cells were then washed twice, adjusted to 5x10^5^ cells/ml and cultured for 72 h at 37°C in Dulbecco´s modified eagle medium (DMEM, Thermo Fisher Scientific, Waltham, USA), supplemented with 10% fetal calf serum (FCS, Biochrom, Berlin, Germany) and 1% penicillin/streptomycin (P/S, Biochrom, Berlin, Germany) supplemented with 100 ng/ml recombinant murine granulocyte colony-stimulating factor (G-CSF, Peprotech, Hamburg, Germany) and 2,5 ng/ml recombinant murine granulocyte-macrophage colony-stimulating factor (GM-CSF, Peprotech, Hamburg, Germany). After 72 h of culture, non-adherent cells were removed and adherent MDSC were detached using 0.5% trypsin/EDTA (Biochrom, Berlin, Germany) and a cell scraper. Purity of Gr 1^+^/CD11b^+^ MDSC was >90%, as determined by flow cytometry.

For induction of T_regs_ by MDSC, *in vitro* generated MDSC from WT and Qa-2^-^ mice were co-cultured with freshly isolated murine CD4^+^ T-cells at a ratio of 2:1 (500 000 T-cells and 250 000 MDSC) in RPMI 1640 with 10% FCS and 1% P/S in 24-well plates at 37°C and 5% CO2. After 3 days of culture, cells were harvested and intracellular Foxp3 staining was performed. CD4^+^ T-cells cultured without MDSC served as control.

For analysis of the effect of anoxia, bacterial stimulation or stimulation with estrogen on Qa-2 expression on MDSC, splenocytes from pregnant WT animals or pregnant HIF-KO animals at E10.5 were isolated and cultured overnight in DMEM with 10% FCS and 1% P/S in 24-well plates at 37°C and 5% CO2. The next day, cells were stimulated for four hours either with anoxia by placing them into a hermetically sealed chamber with anaerobic gas generating sachets (Anaerogen 2.5l, Thermo Fisher Scientific, Waltham, USA), with E. coli at a MOI of 1:50, or with estrogen at concentrations of 1 nM, 10 nM and 100 nM (Merck GmbH, Darmstadt, Germany). After four hours, cells were harvested and extracellular staining was performed.

### Bacterial Culture

E. coli DH5α, an encapsulated K12 laboratory strain was grown in Lennox-Lysogeny-Broth (LB)-medium (Invitrogen, Carlsbad, USA) until early logarithmic growth, resuspended in phosphate buffered saline (PBS, Biochrom, Berlin, Germany) and used immediately.

### T-Cell Suppression Assay

Freshly isolated CD4^+^ splenocytes were stained with carboxyfluorescein-succinimidyl ester (CFSE, Invitrogen, Carlsbad, USA) according to the manufacturer´s instructions. Cells were suspended in RPMI 1640 media containing 1% P/S and 10% FCS. CFSE-labelled CD4^+^ T-cells (2x10^5^) suspended in 100µl media were stimulated with 2x10^5^ mouse T-Activator CD3/CD28 Dynabeads (Thermo Fisher Scientific, Waltham, USA) and 50 ng recombinant murine Interleukin-2 (rmIL-2, R&D Systems, Minneapolis, USA) under addition of ß Mercaptoethanol (Merck, Darmstadt, Germany) at a concentration of 50mM. MDSC isolated from spleens of pregnant WT and Qa-2^-^ animals at E10.5 also suspended in RPMI 1640 containing 1% P/S and 10% FCS were added in different ratios (1:2, 1:4 and 1:8). After 3 days of culture, CD4^+^ T-cell proliferation was determined by flow cytometry using the CFSE dye dilution. Proliferation index, defined as the ratio of CD4^+^ T-cell proliferation after addition of MDSC and CD4^+^ T-cell proliferation without MDSC, was determined. CD4^+^ T-cell proliferation without MDSC was set to a fixed value of 1.

### Flow Cytometry

Human GR-MDSC were characterized as CD66b^+^/CD14^-^/HLA-DR^low/-^ cells, according to previously established protocols for characterization of human MDSC (17). Antibodies used for extracellular staining of human cells were anti-CD66b-FITC (clone G10F, concentration 1 µl/1x10^5^ cells), anti-HLA-G-PE (clone MEM-G/9, concentration 3µl/1x10^5^ cells), anti-HLA-DR-PerCP-Cy5.5 (clone REA805, concentration 0.1 µl/1x10^5^ cells) and CD14-APC (clone MφP9, concentration 1 µl/1x10^5^ cells) [purchased from BD biosciences, Heidelberg, Germany (CD66b and CD14), Miltenyi (HLA-DR) and Exbio, Vestec, Czech Republic (HLA-G)].

For extracellular staining of mouse cells, freshly isolated cells were washed in FACS buffer and fluorescent-conjugated extracellular antibodies were added. Antibodies were purchased from BD Biosciences [CD3 FITC (145-2C11), CD3 PE (17A2), CD4 APC (RM4-5), CD8a APC-H7 (53-6.7), CD8 PE (53-6.7), CD11b Alexa (M1/70), CD19 PE (1D3), CD25 BB515 (PC61), CD44 BB700 (IM7), CD45 BV510 (30-F11), CD45 PerCp (30-F11), CD62L BV421 (MEL-14), CD183 BB700 (CXCR3-173), CD196 BV421 (CCR6), FSV700 Alexa Fluor700, NK1.1 APC (PK136), Gr-1 FITC (RB6-8C5), Gr-1 PerCp (RB6-8C5), Ly-6C FITC (AL-21), Ly-6G PE (1A8)] and Miltenyi [Qa2 PE (REA523)].

For immune cell quantification, cells were pre-gated to CD45. Among CD45^+^ cells, cell types were identified as follows: T-cells CD3^+^, T-Helper cells CD3^+^/CD4^+^, cytotoxic T-cells CD3^+^/CD8^+^, B-cells CD3-/CD19^+^, NK-cells CD3-/NK1.1^+^, MDSC CD11b^+^/Gr-1^+^, MO-MDSC CD11b^+^/Ly6C^+^/Ly6G^-^, GR MDSC CD11b^+^/Ly6Clow/Ly6G^+^ and monocytes CD11b^+^/Gr-1^-^.

For quantification of T-cell subsets cells were pre-gated to CD45, CD3 and CD4 or CD8. Among CD45^+^/CD3^+^/CD4^+^ cells, cell types were identified as follows: naïve T-helper cells CD44^-^/CD62L^+^, effector memory T-helper cells CD44^+^/CD62L^-^, central memory T-helper cells CD44^+^/CD62L^+^, T-helper 1 cells CXCR3^+^/CCR6^-^, T-helper 2 cells CXCR3^-^/CCR6^-^, T-helper 17 cells CXCR3^-^/CCR6^+^, T_reg_ CD25^+^. Among CD45^+^/CD3^+^/CD8^+^ cells, cell types were identified as follows: naïve cytotoxic T cells CD44^-^/CD62L^+^, effector memory cytotoxic T cells CD44^+^/CD62L^-^, central memory cytotoxic T cells CD44^+^/CD62L^+^.

For intracellular staining of Foxp3 cells were extracellular stained with CD4 and CD25 for 30 minutes at 4°C and then incubated in Foxp3 Fixation/Permeabilization working solution (Thermo Fisher Scientific, Waltham, USA) for 60 minutes at room temperature and protected from light. Cells were washed in 1x Permeabilization buffer (Thermo Fisher Scientific) and stained with Foxp3 antibody in 1x permeabilization buffer for 30 minutes at room temperature.

Data acquisition was performed with a FACScalibur or LSR II flow cytometer (BD Bioscience) and analyzed *via* FlowJo V10 (FlowJo, LLC, Ashland, Oregon, USA).

### RNA Isolation and Transcriptome Analyses

For transcriptome analyses of whole uterine lysates, uteri of pregnant WT and Qa 2^-^ mice at E18.5 were collected, snap-frozen in liquid nitrogen and stored at -80°C until RNA isolation. For RNA isolation, frozen tissue (20-30 mg) was shredded using micro pestles (Sigma Aldrich, St.Louis, USA) and liquid nitrogen to obtain powder. RLT buffer was added and the solution was centrifuged at 8000 rcf. RNA isolation was then performed using the RNeasy Mini Kit (Qiagen, Hilden, Germany).

RNA quality was determined by measuring 260/280 and 230/260 absorbance ratio on a spectrophotometer (Nanodrop ND-1000; Peqlab, Erlangen, Germany), RNA concentration was determined using the Qubit Fluorometric Quantitation and RNA Broad-Range Assay (Thermo Fisher Scientific) and RNA Integrity Number RIN using the Fragment Analyzer 5300 and the Fragment Analyzer RNA kit (Agilent Technologies, Santa Clara, USA). For library preparation, mRNA fraction was enriched using polyA capture from 200 ng of total RNA using the NEB Next Poly(A) mRNA Magnetic Isolation Module (New England Biolabs, Frankfurt, Germany). Next, mRNA libraries were prepared using the NEB Next Ultra II Directional RNA Library Prep Kit for Illumina (New England Biolabs) according to the manufacturer’s instructions. Library molarity was determined by measuring the library size (approximately 400 bp) using the Fragment Analyzer 5300 and the Fragment Analyzer DNA HS NGS fragment kit (Agilent Technologies, Santa Clara, USA) and the library concentration (>0.5 ng/µl) using Qubit Fluorometric Quantitation and dsDNA High sensitivity assay (Thermo Fisher Scientific). In the first experiment, libraries were denaturated according to the manufacturer’s instructions, diluted to 270 pM and sequenced as paired-end 100 bp reads on an Illumina NovaSeq 6000 (Illumina) with a sequencing depth >25 million clusters per sample.

Read quality of RNA-seq data in fastq files was assessed using ngs-bits (v.2020_06) to identify sequencing cycles with low average quality, adaptor contamination, or repetitive sequences from PCR amplification. Raw expression values were available for 55.421 genes in 4 samples. Raw gene expression was filtered by demanding a minimum expression value of 1 cpm (counts per million) in at least 2 samples. Filtered data contained expression values for 17.210 genes. Data analysis was performed using the STRING database (40).

### Protein Isolation and Proteome Analyses

For proteome analyses, single cell suspensions were prepared from placentas of pregnant WT and Qa2^-^ animals at E18.5. Cells were lysed by adding lysis buffer [5% 1M Tris/HCl pH 7,4, 2% 5M NaCl, 1% Triton X 100, 1% PMSF, 4% protease inhibitor cocktail (Sigma-Aldrich, St. Louis, USA)] on ice followed by snap-freezing in liquid nitrogen. Ten micrograms of each sample were digested in solution with trypsin as described in (41). After desalting using C18 stage tips, extracted peptides were separated on an Easy-nLC 1200 system coupled to a Q Exactive HFX mass spectrometer (Thermo Fisher Scientific) as described in (42) with slight modifications: The peptide mixtures were separated using a 90 minutes segmented gradient from to 10-33-50-90% of HPLC solvent B (80% acetonitrile in 0.1% formic acid) in HPLC solvent A (0.1% formic acid) at a flow rate of 200 nl/min. The 12 most intense precursor ions were sequentially fragmented in each scan cycle using higher energy collisional dissociation (HCD) fragmentation. Acquired MS spectra were processed with MaxQuant software package version 1.6.7.0 with integrated Andromeda search engine. Database search was performed against a target-decoy Mus musculus database obtained from Uniprot, containing 63.686 protein entries and 286 commonly observed contaminants. Peptide, protein and modification site identifications were reported at a false discovery rate (FDR) of 0.01, estimated by the target/decoy approach. The LFQ (Label-Free Quantification) algorithm was enabled, as well as match between runs and LFQ protein intensities were used for relative protein quantification. Data analysis was performed using the STRING v11 database (40).

### Immunohistochemistry

The mouse placentas at E18.5 were fixed in 4.5% formaldehyde (Sigma Aldrich) and paraffin embedded (max. 48 hours). The samples were infiltrated with paraffin wax in a tissue processor (Leica, Wetzlar, Germany). 3-5 µm thick sections were cut with a sledge microtome (Leica, Wetzlar, Germany) and stained with hematoxylin-eosin (H&E), Periodic acid-Schiff (PAS) and PAS diastase (Merck GmbH, Darmstadt, Germany). Slides were analyzed using an Axioskop 2 plus Zeiss microscope (Zeiss, Oberkochen, Germany) equipped with a Jenoptik ProgRes C10 (Laser Optik System, Jena, Germany) plus camera and software. To quantify the placental phenotype the slides were further evaluated using a histo score with 0 (no aggregates), 1 (intermediate phenotype) and 2 (prominent aggregates) (**Supplementary Figure 5**).

For analysis of spiral artery remodeling at E10.5, uterine arteries were ligated by dental floss and the uterus was removed, placed on a polystyrene piece, fixed in 4.5% formaldehyde (Sigma Aldrich) and paraffin embedded as described in (43). 3-5 µm thick sections were stained with H&E and the slides were scanned with the Ventana DP200 (Roche, Basel, Switzerland). Placentas from all animals were analyzed at the midsagittal point, given by the presence of the chorioallantoic attachment. The total vessel and luminal areas of the spiral arteries were measured in the central 2/4 of the decidua basalis (43). The 5 spiral arteries with the largest and roundest lumen in three consecutive sections (50um between sections) were used for analysis and the mean was calculated.

### Statistical Analysis

Statistical analysis was done using GraphPad Prism 5.0 (GraphPad Software, La Jolla, CA). Data were analyzed for Gaussian distribution using D`Agostino and Pearson omnibus normality test. Unpaired and normally distributed data were analyzed using the unpaired t-test, unpaired and not normally distributed data were evaluated using the Mann-Whitney test. Paired and normally distributed data were analyzed using the paired t-test and paired and not normally distributed data were analyzed using the Wilcoxon matched pairs signed rank test. A p-value <0.05 was considered as statistically significant.

## Results

### Qa-2 Deficiency in Mice Leads to Adverse Pregnancy Outcome

**Figure 1:**
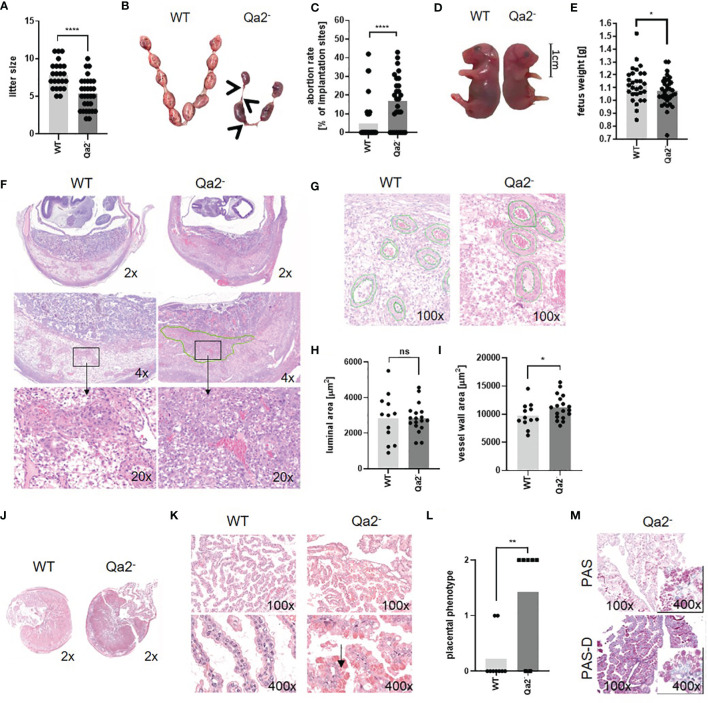
Phenotype of Qa-2^-^ mice during pregnancy. Wildtype (WT) and Qa-2 deficient mice (Qa2^-^) were term-bred and the day when a vaginal plug was detected was defined as day E0.5. Mice delivered spontaneously and litter size was determined **(A)** or mice were euthanized at E18.5 and uteri containing feto-placental units were removed and inspected **(B–E)**. Total implantation sides and resorbing units were counted and fetuses were weighed. **(A)** Litter size of spontaneous delivered WT (n=23) and Qa2^-^ mice (n=30). **(B)** Representative uteri containing feto placental units from WT and Qa-2^-^ mice at gestational day E18.5. Arrows show resorbing units. **(C)** Abortion rate (percentage of resorbed fetuses per litter) of WT (n=39) and Qa-2^-^ mice (n=28) at E18.5. **(D)** Representative WT and Qa-2^-^ fetuses at E18.5. **(E)** Weight of WT (n=29) and Qa-2^-^ fetuses (n=35) at E18.5. **(F–I)** Mice were euthanized at E10.5, uterine arteries were ligated and spiral arteries were analyzed. **(F)** Representative images of H&E stained WT and Qa-2^-^ uteri showing cross-section areas of placenta and decidua in different magnifications. The green line shows an area with unorganized trophoblast distribution within the Qa-2^-^ decidua. **(G)** Representative images of H&E stained WT and Qa-2^-^ uteri showing quantification of spiral artery wall and luminal area. **(H)** Luminal area of spiral arteries from WT (n=5) and Qa-2^-^ animals (n=5). **(I)** Vessel wall area of spiral arteries from WT and Qa-2^-^ animals. **(J–M)** Mice were euthanized at E18.5 and placentas were analyzed. **(J)** Representative images of H&E stained cross-section areas of WT and Qa-2^-^placentas. **(K)** Representative images of H&E stained chorionic villi from WT and Qa-2^-^ placentas showing abnormal vacuoles with eosinophilic aggregates in trophoblasts of Qa-2^-^ animals (arrow). **(L)** Grade of eosinophilic aggregation in trophoblasts of WT and Qa-2^-^ animals. **(M)** Representative images of PAS and PAS-diastase stained chorionic villi from a Qa-2^-^ placenta showing aggregates still present in PAS-diastase staining. Each symbol represents an individual mother **(A, C, L)**/fetus **(E)**/measurement (H+I) and the mean is indicated. ****p < 0.0001; **p < 0.01; *p < 0.05; ns, not significant. Mann-Whitney test **(A, C, L)** or unpaired t-test **(E, H, I)**.

To evaluate the impact of Qa-2 on pregnancy outcome, we analyzed mice lacking the Qa-2 antigen (Qa-2^-^, B6.K1). Compared to WT mice, we found significantly smaller litter sizes in Qa-2- animals (**Figure 1A**). At mid-pregnancy (E10.5), Qa-2^-^ mice had similar numbers of intact fetuses compared to WT mice (**Supplementary Figure 1B**) and slightly increased abortion rates (**Supplementary Figures 1C, D**). At E18.5, lack of Qa-2 led to an abortion rate of 17% in comparison to 5% in WT animals (**Figures 1B, C**) and to an intrauterine growth restriction (IUGR) in surviving fetuses (**Figures 1D, E**).

Since IUGR and late abortions are often a sign of a disturbed blood supply to the fetus, we next analyzed uteri of Qa-2^-^ and WT fetuses histologically. To assess spiral artery remodeling, which is a crucial step in hemodynamic adaptation to pregnancy taking place between E8.5 and E12.5 (43), we analyzed uteri at E10.5. Here, we found profound changes between Qa2^-^ and WT animals; in Qa-2^-^ animals we found large areas within the decidua with unorganized trophoblast distribution, while in WT animals, trophoblasts proper organized around the vessels, pointing to an abnormal trophoblast-migration in Qa-2^-^ mice (**Figure 1F**). In addition, spiral arteries of Qa-2^-^ animals had thicker vessel walls than that of WT mice, while luminal areas did not differ (**Figures 1G–I**). Corresponding to that, we found upregulation of genes encoding for proteins involved in “circulatory system development” in WT uteri in comparison to Qa-2^-^ uteri in transcriptome analyses of whole uterine lysates (**Supplementary Figure 2**).

We further analyzed placenta histology of E18.5 old WT and Qa-2^-^ fetuses; placentas from both genotypes showed similar cross-section areas (**Figure 1J**); however, while placentas from WT animals showed long and thin villi with proper morphology, placentas from Qa-2^-^ animals showed irregular and short villi and abnormal vacuolization of the trophoblast and numerous eosinophilic aggregates. On a scale of 0 (no aggregates) to 2 (prominent aggregates) (**Supplementary Figure 3**), the phenotype of WT placentas was 0.2 ± 0.4 while that of Qa-2^-^ placentas was 1.4 ± 1.0 (**Figures 1K, L**). These aggregates were still present in PAS-diastase staining, indicating that they were not glycogen (**Figure 1M**). Proteome analyses of placenta lysates from WT and Qa-2^-^ animals showed strong enrichment in proteins involved in protein metabolism processes (GO:0019538), especially in translation (GO:0006412), proteolysis (GO:0006508), phosphorylation (GO:0016310) and dephosphorylation (GO:0006470) (**Supplementary Figure 4**) in Qa-2^-^ placentas (184 of 655 proteins only detected in Qa-2^-^), suggesting dysfunctional protein storage in trophoblasts of these animals. On the whole, Qa-2 deficiency led to increased abortions rates in late pregnancy and to disturbed placental vascularization.

### Qa-2 Deficiency Leads to an Altered Immune Cell Composition During Pregnancy

**Figure 2:**
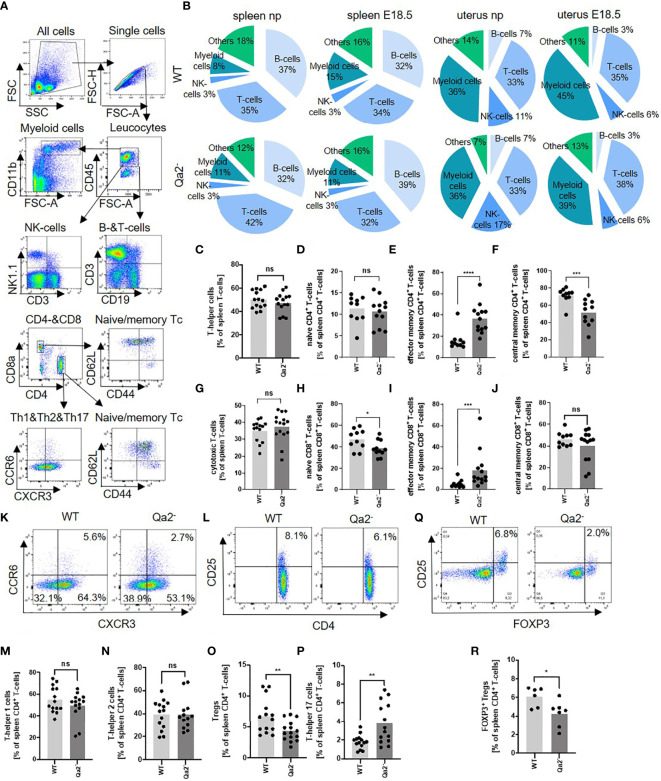
Immune cell composition in WT and Qa-2 deficient animals. Wildtype (WT) and Qa-2 deficient mice (Qa2^-^) were term-bred and the day when a vaginal plug was detected was defined as day E0.5. Spleen cells were analyzed by flow cytometry. **(A)** Gating strategy for gating immune cell subpopulations. **(B)** Proportions of the different cell types in spleens and uteri of non-pregnant (np) and E18.5 pregnant (E18.5) WT (upper diagrams, n=14-17 for np and n=16 for E18.5) and Qa-2^-^ animals (lower diagrams, n=16-21 for npand n=12-13 for E18.5). **(C–J)** Percentages of all T-helper cells **(C)**, naïve T-helper cells **(D)**, effector memory T-helper cells **(E)**, central memory T-helper cells **(F)**, all cytotoxic T-cells **(G)**, naïve cytotoxic T-cells **(H)**, effector memory cytotoxic T-cells **(I)** and central memory cytotoxic T-cells **(J)** from all spleen leucocytes in WT (n=10-14) and Qa-2^-^ animals (n=11-15). **(K)** Representative pseudocolor plots for CXCR3 versus CCR6 showing the populations of T-helper 1 cells (lower right quadrant), T-helper 2 cells (lower left quadrant) and T-helper 17 cells (upper left quadrant) in spleen leucocytes. Cells were pre-gated on CD45, CD3 and CD4. **(L)** Representative pseudocolor plots for CD4 versus CD25 showing the population of T_reg_ cells in spleen leucocytes in the upper right quadrant. Cells were pre-gated on CD45 and CD3. (M-P) Percentages of all T-helper 1 cells **(M)**, T-helper 2 cells **(N)**, T_reg_ cells **(O)** and T-helper 17 cells **(P)** from all spleen leucocytes in WT (n=10-14) and Qa-2^-^ animals (n=11-15). **(Q)** Representative pseudocolor plots for Foxp3 versus CD25 showing the population of Foxp3+ T_reg_ cells in spleen leucocytes in the upper right quadrant. Cells were pre-gated on CD45, CD3 and CD4. **(R)** Percentages of Foxp3 T_reg_ cells from all spleen leucocytes in WT (n=6) and Qa-2^-^ animals (n=7). Each symbol represents an individual animal and the mean is indicated. Light grey bars represent WT animals and dark grey bars represent Qa-2^-^ animals. ****p < 0.0001; ***p < 0.001; **p < 0.01; *p < 0.05; ns, not significant. Mann-Whitney test.

Since it is known that HLA-G plays an important role in immune regulation during pregnancy (44), we next analyzed immune cell populations in spleens and uteri of Qa-2^-^ and WT mice. **Figure 2A** shows gating strategy for immune cell populations. Myeloid cells increased and B-cells decreased in WT spleens and uteri at E18.5 compared to non-pregnant controls while in Qa-2^-^ animals myeloid cells remained unchanged and splenic B-cells even increased. Splenic and uterine T-cell numbers did not change in WT mice during pregnancy but decreased in spleens and increased in uteri of pregnant Qa-2^-^ mice compared to non-pregnant controls. NK-cells decreased in uteri of both WT and Qa-2^-^ animals during pregnancy (**Figure 2B** and **Supplementary Figure 5**). No differences were observed in placental immune cell composition between Qa-2^-^ and WT placentas (**Supplementary Figures 6A–E**).

We next investigated whether there were any differences in T-cell subpopulations between pregnant WT and Qa-2^-^ animals at E18.5. Gating strategy for T-cell subpopulations is depicted in **Figure 2A**, and phenotyping strategy is depicted in **Supplementary Figure 7**. No differences were found in percentages of T-helper cells and cytotoxic T-cells between pregnant WT and Qa-2^-^ animals (**Figures 2C, G**). Qa-2^-^ animals had higher numbers of effector memory CD4^+^ and CD8^+^ T-cells and lower numbers of central memory CD4^+^ and naïve CD8^+^ T-cells (**Figures 2D–F, H–J**). Furthermore, Qa-2^-^ animals had significantly less T_reg_- and more Th17-cells, while there were no differences in numbers of Th1- and Th2-cells (**Figures 2K–P**). Decreased numbers of T_regs_ in Qa-2^-^ animals were confirmed by intracellular staining of FoxP3 (**Figures 2Q, R**).

### Qa-2 Deficiency Leads to Impaired Accumulation and Function of Myeloid Derived Suppressor Cells During Pregnancy

**Figure 3:**
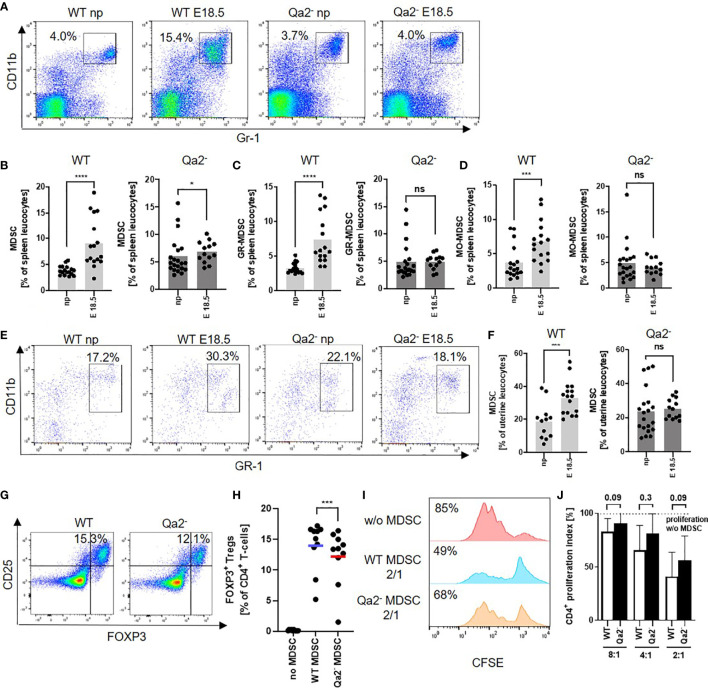
Decreased accumulation and function of MDSC in Qa-2 deficient mice. Wildtype (WT) and Qa-2 deficient mice (Qa2^-^) were term-bred and the day when a vaginal plug was detected was defined as day E0.5. Mice were euthanized at E18.5 and spleens and uteri were collected. Non-pregnant animals served as controls. Tissues were homogenized and filtered to obtain single cell suspensions and cells were analyzed by flow cytometry. **(A)** Representative pseudocolor plots for Gr-1 versus CD11b showing the population of MDSC in spleen leucocytes in the upper right quadrant. Cells were pre-gated on CD45. **(B–D)** Percentages of all MDSC **(B)**, GR-MDSC **(C)** and MO-MDSC **(D)** from all spleen leucocytes in non-pregnant animals (np, n=17 for WT and n=21 for Qa-2^-^) and pregnant animals at E18.5 (n=16 for WT and n=13 for Qa-2^-^). Light grey graphs represent WT animals and dark grey graphs represent Qa-2^-^ animals. **(E)** Representative pseudocolor plots for Gr-1 versus CD11b showing the population of MDSC in uterus leucocytes in the upper right quadrant. Cells were pre-gated on CD45. **(F)** Percentages of MDSC from all uterus leucocytes in non-pregnant animals (np, n=12 for WT and n=20 for Qa-2-) and pregnant animals at E18.5 (n=16 for WT and n=13 for Qa-2^-^). Light grey graph represents WT animals and dark grey graph represents Qa-2^-^ animals. Each symbol represents an individual animal and the mean is indicated. ****p < 0.0001; ***p < 0.001; *p < 0.05; ns, not significant. Mann-Whitney test. MDSC were *in vitro* generated from bone marrow cells (G+H) or isolated by magnetic activated cell sorting from E18.5 pregnant WT and Qa-2^-^ mice (I+J) and added to MACS isolated splenic CD4+ T-cells from WT mice. **(G)** Representative pseudocolor plots for Foxp3 versus CD25 showing the population of Foxp3+ T_reg_ cells after four days of co-culture with MDSC generated from WT and Qa-2^-^ mice in a 2:1 (T-cells: MDSC) ratio in the upper right quadrant. Cells were pre-gated on CD45, CD3 and CD4. **(H)** Percentages of Foxp3^+^ T_reg_ cells of all CD4^+^ T-cells without addition of MDSC, with addition of MDSC generated from WT mice and with addition of MDSC generated from Qa-2^-^ mice (n=10). Each symbol represents an individual animal and the mean is indicated. Bars represent pooled data from 6-7 independent experiments. **(I)** Representative histogram plots showing proliferation of CFSE-stained and anti-CD3/CD28 stimulated T-cells without addition of MDSC and with addition of MDSC generated from WT and Qa-2^-^ mice in a 2:1 (T-cells: MDSC) ratio. **(J)** Inhibitory effect of MDSC from WT mice (white bars) and Qa-2^-^ mice (black bars) on proliferation of CD4^+^ T-cells in different ratios (T-cells:MDSC) (n=6-7). Dashed line shows proliferation of target CD4+ T-cells without addition of MDSC. Proliferation index was determined as ratio of T-cell proliferation with and without addition of MDSC. **p < 0.001; ns, not significant. Wilcoxon matched pairs signed rank test **(H)** and Mann-Whitney test **(J)**.

Since MDSC are critical for maintaining immune tolerance during pregnancy, we investigated whether there were differences in MDSC accumulation and function between WT and Qa-2^-^ animals. We observed a strong increase in total splenic MDSC, as well as in splenic GR-MDSC and MO-MDSC between non-pregnant WT animals and WT animals at E18.5. In Qa-2^-^ animals however, there was only a marginal increase in total splenic MDSC at E18.5, while neither GR-MDSC nor MO-MDSC numbers increased (**Figures 3A–D**). Correspondingly, we found strongly increased numbers of uterine MDSC in WT animals at E18.5 in comparison to non-pregnant controls, but not in Qa-2^-^ animals (**Figures 3E, F**). Interestingly, transcriptome analyses of whole uterine lysates also showed upregulation of genes encoding for proteins involved in “immune system processes” and among that especially in cytokine/chemokine signaling, myeloid cell differentiation, apoptosis regulation, leucocyte migration and lymphocyte activation in WT uteri in comparison to Qa-2^-^ uteri (**Supplementary Figure 2**).

We further analyzed the functional capacity of MDSC from WT and Qa-2^-^ animals and found a decreased capacity to induce T_regs_ *in vitro* (**Figures 3G, H**) and a slightly but not significantly reduced capacity of Qa-2^-^ MDSC to inhibit T-cell proliferation in comparison to WT MDSC (**Figures 3I, J**).

### Expression of Qa-2 on MDSC Is Regulated by Estrogen *via* HIF-1α

**Figure 4:**
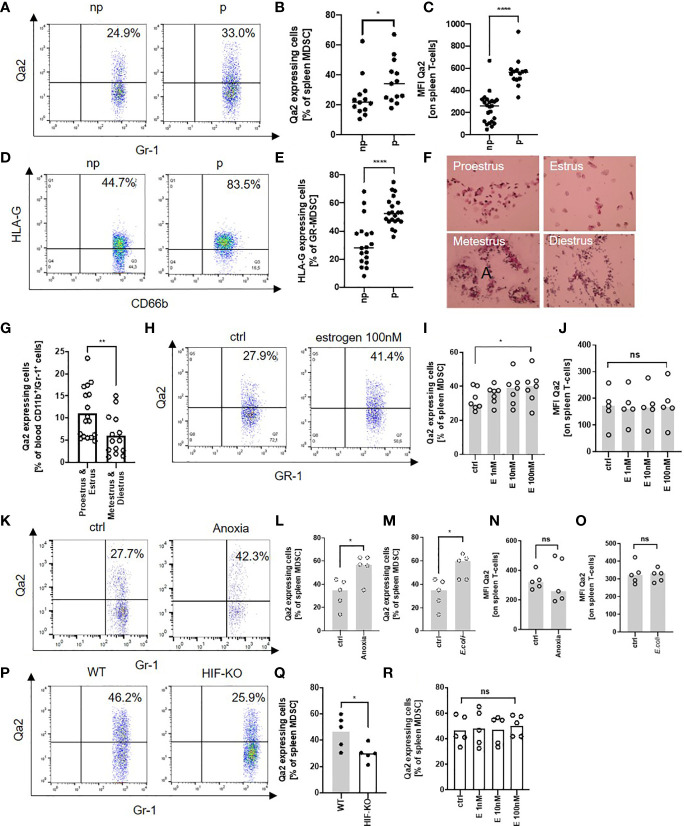
Expression of Qa-2 on MDSC is regulated by estrogen *via* HIF-1α. **(A)** Representative pseudocolor plots for Gr-1 versus Qa-2 from splenocytes from non-pregnant (np) and pregnant (p) wildtype (WT) animals showing the percentage of Qa-2 expressing MDSC in the upper right quadrant. Cells were pre-gated on CD45 and CD11b. **(B)** Percentages of Qa-2 expressing MDSC from all spleen MDSC from non-pregnant (n=13) and pregnant WT mice (n=13). **(C)** MFI for Qa-2 on spleen T-cells from non-pregnant (n=23) and pregnant WT mice (n=14). **(D)** Representative pseudocolor plots for CD66b versus HLA-G from PBMC from non-pregnant (np) and pregnant (p) women showing the percentage of HLA-G expressing MDSC in the upper right quadrant. **(E)** Percentages of HLA-G expressing MDSC from all MDSC in the peripheral blood of non-pregnant (n=18) and pregnant women (n=21). **(F)** Representative images of H&E stained vaginal swabs from the four different phases of the mouse estrus cycle. **(G)** Percentages of Qa-2 expressing cells from all blood CD11b^+^/Gr-1^+^ cells in proestrus & estrus (n=14) and metestrus & diestrus (n=17). **(H)** Representative pseudocolor plots for Gr-1 versus Qa-2 from splenocytes from WT mice without stimulation (ctrl) and with stimulation with 100nM estrogen showing the percentage of Qa-2 expressing MDSC in the upper right quadrant. Cells were pre-gated on CD45 and CD11b. **(I)** Percentages of Qa-2 expressing MDSC from all spleen MDSC from WT mice without stimulation and with stimulation with estrogen in rising concentrations (n=7). **(J)** MFI for Qa-2 on spleen T-cells from wildtype mice without stimulation and with stimulation with estrogen in rising concentrations (n=5). **(K)** Representative pseudocolor plots for Gr-1 versus Qa-2 from splenocytes from wildtype (WT) mice cultured under normoxia (ctrl) or anoxia for 4 h showing the percentage of Qa-2 expressing MDSC in the upper right quadrant. Cells were pre-gated on CD45 and CD11b. **(L)** Percentages of Qa-2 expressing MDSC from all spleen MDSC from WT mice cultured under normoxia or anoxia (n=5). **(M)** Percentages of Qa-2 expressing MDSC from all spleen MDSC from WT mice without stimulation or with stimulation with **(E)** coli (n=5). **(N)** MFI of Qa-2 on spleen T-cells from WT mice cultured under normoxia or anoxia (n=5). **(O)** MFI of Qa-2 on spleen T-cells from wildtype mice without stimulation or with stimulation with **(E)** coli (n=5). **(P)** Representative pseudocolor plots for Gr-1 versus Qa-2 from splenocytes from pregnant WT animals and pregnant animals with targeted deletion of HIF-1α in myeloid cells (HIF-KO) showing the percentage of Qa-2 expressing MDSC in the upper right quadrant. Cells were pre-gated on CD45 and CD11b. **(Q)** Percentages of Qa-2 expressing MDSC from all spleen MDSC from WT mice (n=5) and from HIF-KO mice (n=5). **(R)** Percentages of Qa-2 expressing MDSC from all spleen MDSC from HIF-KO mice without stimulation and with stimulation with estrogen in rising concentrations (n=5). Each symbol represents an individual animal/women and the mean is indicated. ****p < 0.0001; **p < 0.01; *p < 0.05; ns, not significant. Mann-Whitney test **(B, C, E, G, Q)** and Wilcoxon matched-pairs signed rank test **(I, J, L–O, R)**.

As Qa-2^-^ MDSC had reduced functional capacity, we next asked how Qa-2^-^ expression may be regulated. Flow cytometric analyses of Qa-2 expression on MDSC and T-cells revealed that in non-pregnant WT mice between 10% and 60% of MDSC and all T-cells expressed Qa-2, however Qa-2 expression on MDSC (MFI mean 33.8 ± 25.8) was much lower than on T-cells (MFI 241.7 ± 137.4); pregnancy increased the expression of Qa-2 on both MDSC and T-cells (**Figures 4A–C**). The same effect could be observed for HLA-G-expression on human MDSC (**Figures 4D, E**). To get hints on a potential hormonal regulation of Qa-2 expression on immune cells, we analyzed blood of female mice during the menstrual cycle and found increased Qa-2 expression on MDSC during proestrus and estrus, the phases with higher estrogen levels (23), than during metestrus and diestrus (**Figures 4F, G**). To further evaluate the effect of estrogen on Qa-2 expression on MDSC, we next stimulated spleen cells of WT mice with increasing concentrations of estrogen and showed that Qa-2 expression on MDSC increased upon estrogen stimulation in a concentration dependent manner, while Qa-2 expression on T-cells did not change (**Figures 4H–J**). Recent data showed that expression of HLA-G on tumor cells can be regulated by the transcription factor hypoxia-inducible factor 1α (HIF-1α) (45, 46) and that HIF-1α regulates MDSC function during murine pregnancy (31). We thus assumed that expression of Qa-2 on MDSC may be regulated by HIF-1α and stimulated spleen cells of WT mice with classic (anoxia) and alternative (Escherichia coli, E. coli) stimuli of HIF-1α. We found that both anoxia and E. coli stimulation led to an increased expression of Qa-2 on MDSC (**Figures 4K–M**), but not on T-cells (**Figures 4N, O**). Correspondingly, MDSC isolated from pregnant mice with targeted deletion of HIF-1α in myeloid cells (HIF-KO) expressed lower levels of Qa-2 than MDSC isolated from WT mice (**Figures 4P, Q**). Stimulation of myeloid HIF-KO MDSC with estrogen did not result in an upregulation of Qa-2 expression (**Figure 4R**). Taken together, our results show that expression of Qa-2 on MDSC is regulated by estrogen *via* HIF-1α.

### Application of sHLA-G Improves Pregnancy Outcome via MDSC

**Figure 5:**
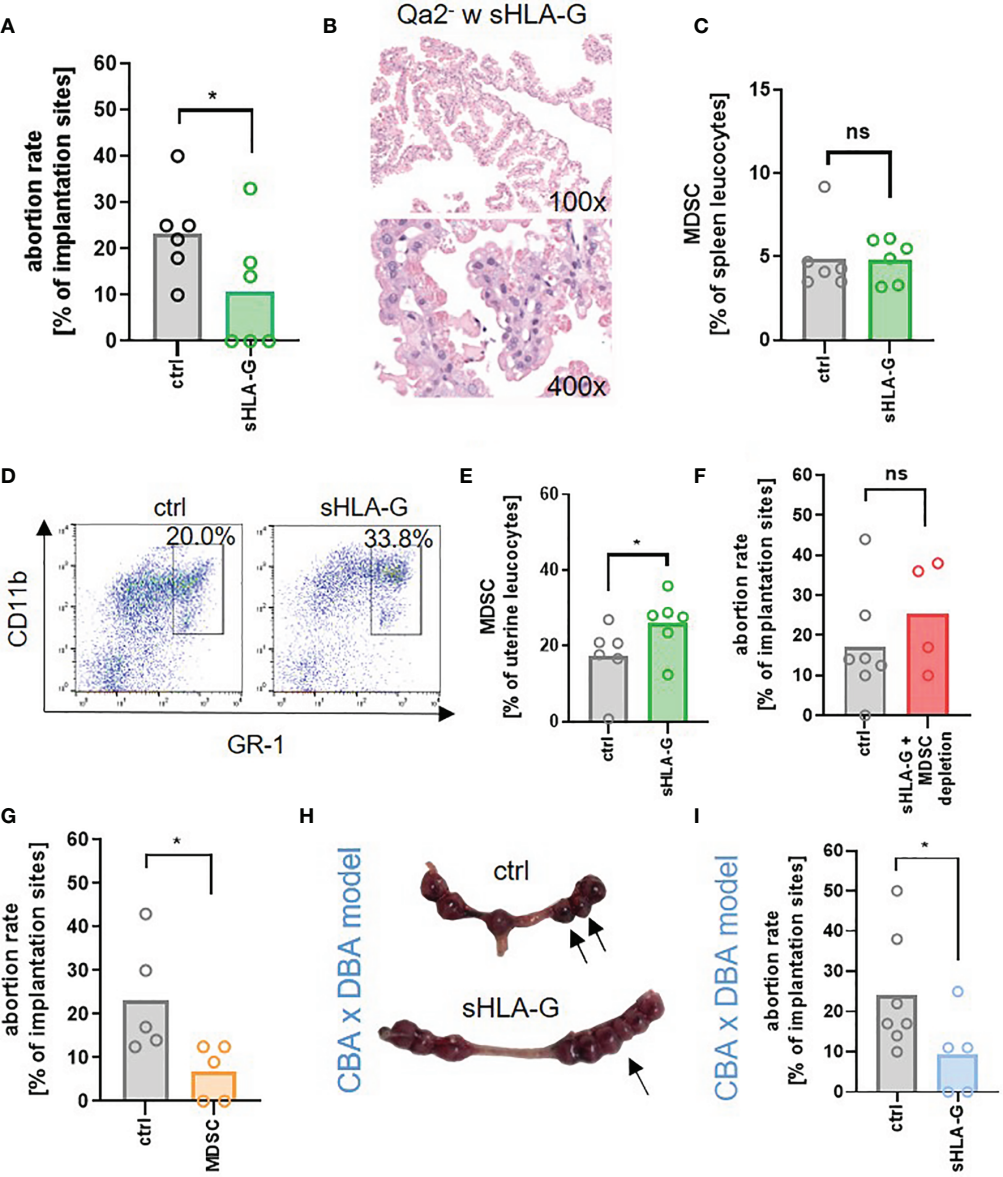
sHLA-G improves pregnancy outcome. **(A–F)** Qa-2 deficient mice (Qa2^-^) were term-bred and the day when a vaginal plug was detected was defined as day E0.5. At E10.5 and E14.5 mice received either PBS, 1µg/g bodyweight sHLA-G or 1µg/g bodyweight sHLA-G and anti-Ly6G-antibody. **(A)** Abortion rate (percentage of resorbed fetuses per litter) of Qa-2^-^ mice after application of PBS (ctrl, n=6) or sHLA-G (n=6) at E18.5. **(B)** Representative images of H&E stained chorionic villi Qa-2^-^ animals that received sHLA-G. **(C)** Percentages of MDSC in spleens of pregnant Qa-2- animals after application of PBS (ctrl, n=6) or sHLA-G (n=6) at E18.5. **(D)** Representative pseudocolor plots for Gr-1 versus CD11b showing the population of MDSC in uterus leucocytes of Qa-2^-^ mice after application of PBS or sHLA-G in the upper right quadrant. Cells were pre-gated on CD45. **(E)** Percentages of MDSC in uteri of pregnant Qa-2^-^ animals after application of PBS (ctrl, n=6) or sHLA-G (n=6) at E18.5. **(F)** Abortion rate of Qa-2 deficient mice after application of PBS (ctrl, n=7) or sHLA-G and anti-Ly6G-antibody (n=4) at E18.5. (G+H) Female CBA/J mice were term-bred with male DBA/2J mice and the day when a vaginal plug was detected was defined as day E0.5. At E0.5, E3.5, E6.5 and E9.5 mice received either PBS or 1µg/g bodyweight sHLA-G. **(G)** Abortion rate of Qa-2 deficient mice after application of PBS (ctrl, n=5) or MDSC (n=5) at E18.5. (H+I) Female CBA/J mice were term-bred with male DBA/2J mice and the day when a vaginal plug was detected was defined as day E0.5. At E0.5, E3.5, E6.5 and E9.5 mice received either PBS or 1ug/g bodyweight sHLA-G. **(H)** Representative uteri containing fetal-placental units from PBS (ctrl, n=7) and sHLA-G treated mice (n=5) at gestational day E10.5. Arrows show resorbed fetuses. **(I)** Abortion rate of CBA/J mice after application of PBS (ctrl, n=7) or sHLA-G (n=5) at E10.5. Each symbol represents an individual animal and the mean is indicated. *p < 0.05; ns, not significant. Mann-Whitney test.

Lastly, we asked whether we could restore pregnancy success in Qa-2^-^ animals by application of sHLA-G. Pregnant Qa-2^-^ mice received either 1µg/g bodyweight sHLA-G or PBS at E10.5 and E14.5. Application of sHLA-G led to a pronounced reduction in the abortion rate of Qa-2^-^ animals (**Figure 5A**), accompanied by a partial restoration of normal trophoblast morphology (**Figure 5B**). Furthermore, it marginally increased splenic MDSC, but strongly increased uterine MDSC (**Figures 5C–E**). Simultaneous depletion of MDSC with sHLA-G application reversed the pregnancy-protective effect of sHLA-G (**Figure 5F**). Conversely, adoptive transfer of *in vitro* generated MDSC also resulted in a reduced abortion rate in Qa-2^-^ animals (**Figure 5G**). To confirm the beneficial effect of sHLA-G on pregnancy outcome in another model, we treated abortion-prone DBA/2J-mated CBA/J mice (suffering from early abortions) with sHLA-G or PBS. Correspondingly, in this model, application of sHLA-G significantly reduced abortion rates (**Figures 5H, I**).

## Discussion

Our data show that the murine MHC-Ib molecule Qa-2 is relevant for pregnancy success and protects from late pregnancy loss by regulating immune adaptation to pregnancy in terms of modulating T-cell homeostasis and promoting MDSC accumulation. We further show that expression of Qa-2 on MDSC is relevant for their functionality and regulated by estrogen *via* HIF-1α. Lastly, we show that application of sHLA-G to abortion-prone Qa-2^-^ mice decreases abortion rates *via* induction of MDSC. Adoptive transfer of *in vitro* generated MDSC had the same effect as HLA-G application.

It has been shown for a long time that HLA-G is highly expressed during pregnancy especially by trophoblast cells (3) mediating various immune-modulatory effects *in vitro* (4–6). Furthermore, alterations of HLA-G expression during pregnancy are associated with adverse pregnancy outcome such as preeclampsia (47–50). Until now, however, little *in vivo* evidence for a pregnancy-preserving role of HLA-G exists. Since Qa-2 is the most similar murine MHC-Ib molecule to human HLA-G described so far, we used Qa-2 deficient mice to evaluate the *in vivo* role of Qa-2 and HLA-G for pregnancy outcome.

Our first result of smaller litter sizes and growth restriction in surviving fetuses in Qa-2^-^ animals in comparison to WT animals confirms previous studies also describing smaller litters and smaller offspring in Qa-2^-^ animals (20, 25) and studies in humans showing an association of HLA-G gene polymorphisms and birth weight (21). In murine studies, expression of Qa-2 on the fetal side was found to be advantageous for survival, leading to a higher embryonic cleavage rate (20, 51, 52). Correspondingly, HLA-G expression in early embryos seems to be important for obtainment of pregnancy (18, 19) and maternal expression and haplotype of HLA-G may predict success of *in vitro* fertilization (53). Additionally, we now show increased rates of pregnancy loss in Qa-2^-^ animals especially during late pregnancy accompanied by profound changes in maternal adaptation to pregnancy in comparison to WT mice, demonstrating that Qa-2 not only plays a local role in fetal tissue but is also systemically needed in the maternal organism to facilitate a successful pregnancy.

In pregnant Qa-2^-^ animals we found an unorganized trophoblast invasion and altered spiral artery morphology with thicker vessel in comparison to WT animals. Remodeling of uterine spiral arteries driven by invasion of trophoblast cells to the maternal decidua is one of the critical steps in maternal adaptation to pregnancy as it permits normal placental perfusion and fetal growth and development (54). Inadequate trophoblast invasion and spiral artery remodeling results in placental hypoxia and may lead to development of preeclampsia and fetal growth restriction (55). It should be mentioned at this point that the structure of human and murine placentas and thus also the localisation of HLA-G and Qa2 differ. HLA-G is expressed by extravillous trophoblast cells, which migrate into the maternal decidua, whereas Qa-2, which is expressed by trophoblast giant cells, is found more locally at the boundary layer between decidua and labyrinth (56, 57).

In Qa-2^-^ placentas, we observed profound changes in trophoblast morphology in comparison to WT placentas with cytoplasmic storage of eosinophilic aggregates and enrichment of proteins involved in protein metabolism. Interestingly, it has been shown that during preeclampsia, misfolded proteins accumulate in urine, serum and placenta similar to the protein accumulation observed in neurodegenerative disorders like Alzheimer´s disease (58–60). Our findings of an altered trophoblast invasion and spiral artery remodeling, pathological protein storage in placenta, fetal growth restriction and late abortions may suggest the development of a preeclampsia-like phenotype in Qa-2^-^ mice. This assumption is supported by recent data from others showing that injection of an anti-Qa-2 antibody led to preeclampsia symptoms in mice that could be abrogated by simultaneous injection of recombinant VEGF (61). It also corresponds to human data showing that an altered pattern of HLA-G expression on trophoblast cells is associated with preeclampsia (47, 50).

We further found significant differences in immunological adaptation to pregnancy between WT and Qa-2^-^ animals. In pregnant Qa-2^-^ mice but not in pregnant WT mice we found an increase in splenic T-cells in comparison to non-pregnant controls – an effect that may be associated with pregnancy loss (62). Analysis of T-cell subpopulations showed differences in numbers of effector memory T-cells, T_regs_ and Th17 cells between pregnant WT and Qa-2^-^ animals. The CD44^+^/CD62L- effector memory CD4^+^ and CD8^+^ T-cell subsets were increased in pregnant Qa-2^-^ mice, while naïve CD8^+^ and central memory CD4^+^ T-cells were decreased. This points towards a higher activation status of T-cells in Qa-2^-^ mice. A recent study showed that effector/activated T-cells led to adverse pregnancy outcome, i.e. preterm birth (63). Furthermore, patients with preeclampsia downregulate CD62L on T-cells (64).

Balance between Th1 and Th2 cells did not differ between WT and Qa-2^-^ animals, while T_regs_ decreased and Th17 cells increased in pregnant Qa-2^-^ in comparison to pregnant WT mice. This effect corresponds to the T-helper cell changes described in patients with preeclampsia (65). Studies in mice showed that expansion of T_regs_ was relevant for healthy pregnancy and that adoptive transfer of T_regs_ protected from abortions (66, 67).

Both, systemically and locally in the uterus, we observed a strong increase in MDSC in WT but not in Qa-2^-^ mice. The accumulation of MDSC and its relevance for successful pregnancy has been described previously in different mouse models [reviewed in (68)]. However, all these studies focused on early- to mid-gestation (31–33). Our present results now illustrate that MDSC accumulation also seems to be relevant for pregnancy success in later stages of pregnancy. Interestingly, Ostrand-Rosenberg et al. showed that a single depletion of MDSC at E8.5 did not affect pregnancy outcome (32). However, since antibody-mediated MDSC depletion lasts only three days (69), one injection at E8.5 may be insufficient to examine the role of MDSC during late pregnancy. In correspondence to the increased accumulation of MDSC in uteri of WT mice we observed an upregulation of genes involved in myeloid differentiation, leucocyte migration and chemotaxis in transcriptome analyses of WT whole uterine lysates in comparison to Qa-2^-^ animals. Corresponding to that, we previously showed that sHLA-G *in vitro* led to a quantitative and functional induction of MDSC (34). We thus assume that a combination of direct and indirect effects of Qa-2 attract MDSC to the pregnant uterus *in vivo*.

The exact mechanism by which Qa-2 interacts with MDSC during pregnancy remains unclear. There are two candidates for Qa-2 receptors on immune cells, the paired immunoglobulin-like inhibitory receptor (PIR-B) on myeloid cells and B-cells, which have been shown to interact with MHC class I (70) molecules and HLA-G (71, 72) and Ly49C on NK-cells (73). However, for both, clear evidence for a functional interaction with Qa-2 is lacking. MDSC from pregnant WT animals expressed PIR-B (data not shown) and therefore may be able to bind Qa-2/HLA-G. However, we did not go further into detail of this interaction for example by simultaneous blocking PIR-B and application of HLA-G.

Since the main effector function of MDSC is an inhibition of T-cell activation, one could hypothesize that increased activation of T-cells in Qa-2^-^ animals results from a decreased MDSC influence. However, contrary to our results, MDSC have been shown to downregulate CD62L on T-cells (32, 74). As Qa-2 is also highly expressed on T-cells, the lack of Qa-2 itself may lead to differences in T-cell activation between WT and Qa-2^-^ mice overlapping the effect of MDSC.

A crosstalk between T_regs_ and MDSC has been described extensively under tumor conditions (reviewed in (75)). Kang et al. described an induction of T_regs_ by MDSC *via* production of TGF-β *in vivo* during pregnancy (76), while we showed *in vitro* that fetal human MDSC as well as exosomes released by MDSC from pregnant women were able to induce T_regs_ (77, 78). We now demonstrate that induction of T_regs_ by MDSC was reduced in absence of Qa-2 on MDSC. This is in line with two studies showing an induction of T_regs_ by mesenchymal stem cells (MSCs) *via* HLA-G (79). T_reg_ induction by MDSC may explain the decreased numbers of T_regs_ in pregnant Qa-2- mice. Although the differences we measured in our functional assay are not huge, together with the lower Treg levels in pregnant Qa-2^-^ mice, we consider this effect to be biologically relevant.

Due to the observed functional differences between WT and Qa-2^-^ MDSC, we asked whether Qa-2 contributes to the functional activation of MDSC during pregnancy. Although the main source of Qa-2 during pregnancy is embryonic tissue, it is known that Qa-2 is also expressed on immune cells (36). We found an obvious, albeit low, expression of Qa-2/HLA-G also on MDSC. Furthermore, we found that MDSC isolated from pregnant individuals (mice and women) expressed higher levels of Qa-2, respectively HLA-G, than MDSC from non-pregnant individuals and that Qa-2 expression on MDSC, but not on T-cells, could be stimulated by the pregnancy hormone estrogen. Immunomodulatory effects of estrogens have been repeatedly described, e.g. an expansion of T_regs_ and a modulation of Th-cell cytokine expression (80, 81). Furthermore, it could be shown that estrogen mediates expansion and functional activation of MDSC (82, 83). However, upregulation of Qa-2 expression on MDSC by estrogen is a yet unknown mechanism.

We further showed that the effect of estrogen on Qa-2 expression on MDSC during pregnancy was mediated through HIF-1α. This is in line with results from other groups showing that estrogen can activate HIF-1α (84) and that activation of HIF-1α stimulated HLA-G-expression in cancer cells (45, 46). We recently showed that expression of HIF-1α was relevant for MDSC accumulation and function during pregnancy and that targeted deletion of HIF-1α in myeloid cells (myeloid HIF-KO) led to pregnancy failure in terms of abortions (31). Our new results suggest that an impaired expression of Qa-2 on MDSC may at least partially be responsible for the adverse pregnancy outcome in myeloid HIF-KO mice.

Lastly, we aimed to investigate the therapeutic effect of sHLA-G on pregnancy outcome. We showed that application of sHLA-G reduced the abortion rate in Qa-2^-^ animals, restored placental morphology and induced uterine MDSC accumulation. Simultaneous antibody-mediated depletion of MDSC nullified the protective effect of sHLA-G while adoptive transfer of *in vitro* generated MDSC protected from abortions as well as HLA-G application. Interestingly, the protective effect of sHLA-G on abortion rate was also observed in a mouse model with early abortions (CBA/J x DBA/2J mating model) pointing towards positive effects not only during late but also during early gestation. The fact that CBA/J mice also lack Qa-2 expression may explain their good response to HLA-G. Previous studies in mice showed protective effects of HLA-G on transplant rejection (71, 85) and collagen-induced arthritis (86); furthermore, sHLA-G was shown to allow tumor evasion from immunosurveillance (72, 87), with some of these effects being mediated by an expansion of MDSC (72, 87, 88). These results together with those reported here suggest that a mutual support of HLA-G and MDSC helps to protect allografts from immune rejection and that this interaction is helpful whenever tolerance is needed to survive (pregnancy, organ transplantation), but detrimental in case of tumor growth. Further studies are needed to work out the exact mechanisms behind this effect at different stages of pregnancy. As we aimed to demonstrate an *in vivo* protective effect of HLA-G on murine pregnancy outcome that could be transferred to human pregnancy, we did not include the application of recombinant Qa-2 in our experimental settings.

One limitation of our study is that we used a syngeneic mating model. Since allo antigens play an important role for immunological pregnancy complications and especially for preeclampsia it may be worth to investigate allogeneic pregnancy. However, in our case, allogeneic mating would have led to expression of Qa-2 by the fetuses making it impossible to investigate the effect of a total lack of Qa-2. Another limitation is that HLA-G is not endogenously expressed in mice. Thus, we used mice lacking Qa-2, the only homologue-candidate for HLA-G yet known, for analyzing its impact on pregnancy outcome. However, although HLA-G is a human MHC I molecule, it binds to the murine paired immunoglobulin-like inhibitory receptor (PIR-B) and mediates tolerogenic effects in mice making it possible to analyze its effects *in-vivo* (71, 72). As we observed relevant pregnancy losses in our model in the second half of pregnancy, we started HLA-G application only on day E10.5. Earlier HLA-G administration could have an additional positive effect, as it could possibly already influence the spiral artery remodeling in the uterus at earlier timepoints. Another point worth mentioning is that, in contrast to previous studies with Qa-2 mice (B6.K1) (24, 25), we did not use B6.K2 as a control strain, but normal C57BL6/J mice. To our best knowledge and the knowledge of the supplier, the only difference between B6.K2 and C57BL6/J is that B6.K1 and B6.K2 models were developed in parallel and B6.K2 mice might differ from C57BL6/J mice in the substrain. However this does not influence the haplotype, which is H2^b4^ for both B6.K2 and C57BL6/J (carrying the allels K^b^, D^b^, Qa 2^a(hi)^ and Tla^b^) and thus should not influence pregnancy outcome.

In conclusion, we here describe the impact of Qa-2 on maternal phenotype and immune adaptation during pregnancy, providing evidence that Qa-2 may prevent the development of preeclampsia and abortions by promoting MDSC accumulation and functional activation. We further show for the first time *in-vivo* that application of sHLA-G improves pregnancy outcome *via* MDSC induction. These results give reason to hope that synthetic sHLA-G may find a place in the prevention of immunological pregnancy complications like abortions and preeclampsia.

## Data Availability Statement

The datasets presented in this study can be found in online repositories. The names of the repository/repositories and accession number(s) can be found below: https://www.ncbi.nlm.nih.gov/geo/, accession ID: GSE186053.

## Ethics Statement

The studies involving human participants were reviewed and approved by Ethikkommission an der Medizinischen Fakultät der Eberhard-Karls-Universität Tübingen und am Universitätsklinikum Tübingen. The patients/participants provided their written informed consent to participate in this study. The animal study was reviewed and approved by Regierungspräsidium Tübingen.

## Author Contributions

SD, JS, AV, IG-M, LQ-M, NC, AM, CG, and NK-G contributed to the acquisition of data, analysis, and interpretation of data. CP provided critical feedback on intellectual content. NK-G and CG conceived the study and wrote the paper. NK-G and CG contributed equally. All authors were involved in drafting the article or revising it critically for important intellectual content, and all authors approved the final version to be published.

## Funding

This work was supported by research grants of the Ministerium für Wissenschaft, Forschung und Kunst Baden-Württemberg and the European Social Fund and the Deutsche Forschungsgemeinschaft (DFG) (Grants no. GI 1094/4-1 and GI 1094/4-2).

## Conflict of Interest

The authors declare that the research was conducted in the absence of any commercial or financial relationships that could be construed as a potential conflict of interest.

## Publisher’s Note

All claims expressed in this article are solely those of the authors and do not necessarily represent those of their affiliated organizations, or those of the publisher, the editors and the reviewers. Any product that may be evaluated in this article, or claim that may be made by its manufacturer, is not guaranteed or endorsed by the publisher.
