# Supplementary material for: Human Leucocyte Antigen G and Murine Qa-2 Are Critical for Myeloid Derived Suppressor Cell Expansion and Activation and for Successful Pregnancy Outcome
Source: Front Immunol. 2022 Jan 17;12:787468. doi: 10.3389/fimmu.2021.787468 (PMC8801456; doi:10.3389/fimmu.2021.787468)
Supplement: Supplementary file 1 [file DataSheet_1.pdf]

Supplementary Figure 1

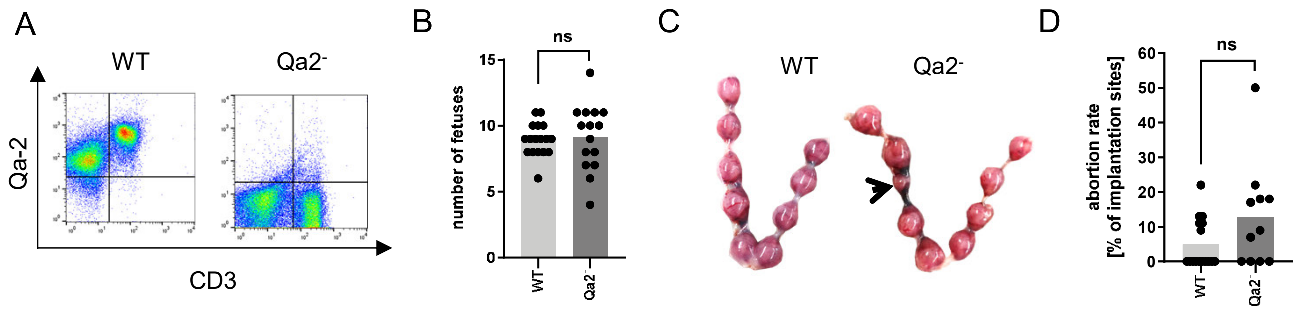

Supplementary Figure 1: Number of fetuses and abortion rate in Qa-2$^-$ mice at E10.5

(A) Representative densitiy plots for CD3 versus Qa-2 show expression of Qa-2 on spleen leucocytes of wildtype (WT) and Qa-2 deficient mice (Qa2$^-$). (B-D) Wildtype (WT) and Qa-2 deficient mice (Qa2$^-$) were term-bred and the day when a vaginal plug was detected was defined as day E0.5. Mice were euthanized at E10.5 and uteri containing feto-placental units were removed and inspected. Total implantation sides and resorbing units were counted. (B) Number of intact fetuses of WT (n=17) and Qa2$^-$ mice (n=15). (C) Representative uteri containing feto-placental units from WT and Qa2$^-$ mice at gestational day E10.5. Arrows show resorbing units. (D) Abortion rate (percentage of resorbed fetuses per litter) of WT (n=16) and Qa2$^-$ mice (n=11) at E10.5. Each symbol represents an individual animal and the mean is indicated. ns = not significant. Unpaired t-test (A) or Mann-Whitney test (B).

Supplementary Figure 2

A

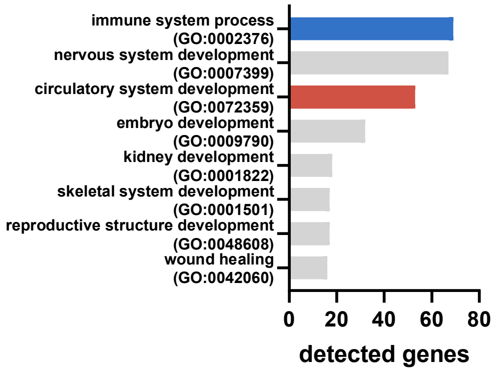

B

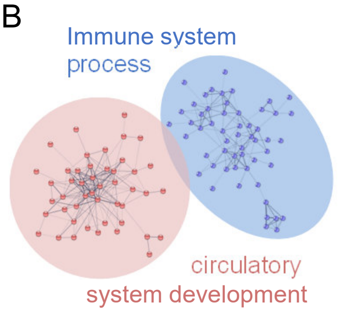

Biological process	Gene names
**Blood vessel developme nt**	Adm, Angpt2, Bax, Cdh5, Col8a1, Dll4, Ecscr, Ednra, Egfl7, Ephb4, Ets1, Fgf1, Flt4, Foxc2, Gja4, Gja5, Gpr116, Hey1, Itgb1bp1, Kdr, Loxl2, Mmrn2, Nos3, Nrcam, Pdgfb, Pecam1, Pgf, Plau, Prox1, Ptprb, Rasip1, S1pr1, Sema3e, Sox18, Spi1, Tal1, Tbx1, Tek, Tgfb1, Thbs1, Tie1, Tspan12, Unc5b
**others**	Smad9, Atoh8, Ets1, Cld5, Oxtr, Ednra, Nrgn, Sema3e

Biological process	Gene names
**cytokine/ chemokine signaling**	Ccl21a, Ccl8, Ccl9, Ccl6, Cxcl1, H2-Q7, Fcer1g, Tnfrsf9, Fas, Gas6, Il3ra, Pdgfb, Ccrlf2, Ets1, Tnfrsf11b, Ifnar2, Il11ra, Isg15, Ifit1, Ifit2, Ifit3, Iigp1, Cxcl14
**myeloid cell differentiation**	Tyrobp, Fcer1g, Spi1, Fli1, Tgfb1, Tal1, Hbb-bt, Hba-a1, Alas2, Dmtn, Gpr116
**apoptosis regulation**	Bax, Ccl21a, Tnfrsf9, Fs, Fcer1g, Gas6, Kdr, Tek, Tbx1, Pdpn, Tgfb1, Inhba, Ets1, Tnsfrsf11b, Ifit2, Ifit3
**leucocyte migration**	Ccl21a, Il17b, Cxcl1, Ccl9, Ccl8, Ccl6, S1pr1, Pecam1, Gas6, Fcer1g, Pdgfb, Podoxl
**lymphocyte activation**	Bax, Ccl21a, Spi1, Fas, Fcer1g, Dll4, Tgfb1, Il11ra, Rsad2, CD80
**others**	Ptx3, Meox1, C1qa, C1qb, H2-Q6, Siglecg, Angpt2, Lmo2

Supplementary Figure 2: Transcriptome analyses of uteri from wildtype and Qa-2 deficient mice

Uteri from wildtype (WT, n=2) and Qa-2 deficient mice (Qa-2^{-}, n=2) were collected at E18.5 of pregnancy and total RNA was isolated and sequenced. (A) Selected biological processes enriched in WT uteri in comparison to Qa2^{-} uteri. (B) Tables of single genes upregulated in WT uteri in comparison to Qa2^{-}uteri assigned to the biological processes "immune system processes" (blue) and "circulatory system development" (red).

Supplementary Figure 3

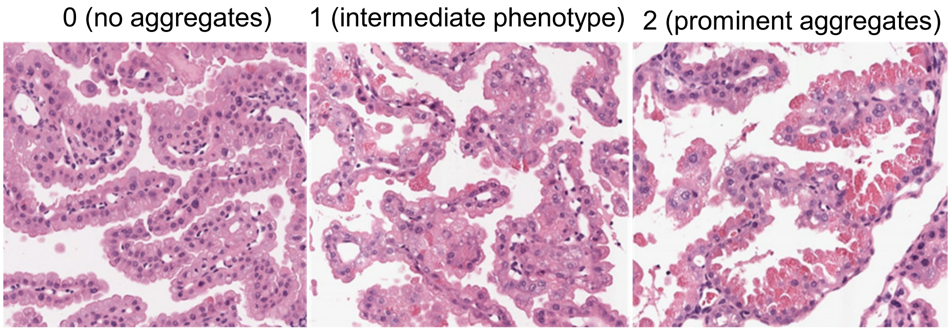

Supplementary Figure 3: Score for analysis of placental phenotype

Wildtype (WT) and Qa-2 deficient mice (Qa2-) were term-bred and the day when a vaginal plug was detected was defined as day E0.5. Mice were euthanized at E18.5 and placentas were removed and analyzed by immunohistochemistry. Representative images of H&E stained cross-section areas of placentas shows grading of eosinophilic aggregation in trophoblasts.

Supplementary Figure 4

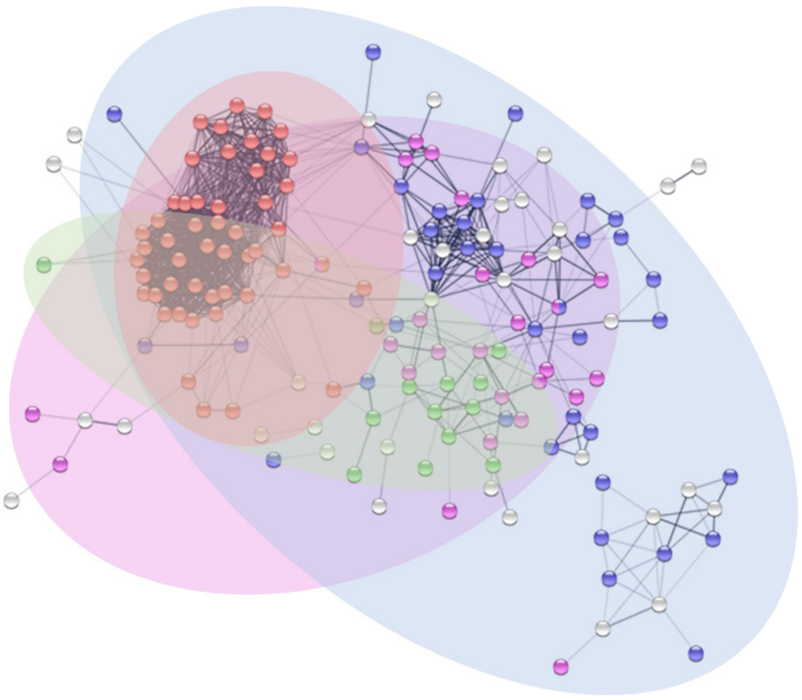

Supplementary Figure 4: Proteome analyses of placentas from wildtype and Qa-2 deficient mice

Uteri from wildtype (WT, n=3) and Qa-2 deficient mice (Qa-2^{-}; n=3) were collected at E18.5 of pregnancy, single cell suspensions were prepared and total protein was isolated analysed by mass spectometry. Network analysis shows proteins only expressed in Qa-2^{-} placentas involved in protein metabolism processes (red – translation; blue – proteolysis; purple – phosphorylation; green – dephosphorylation; grey – others).

Supplementary Figure 5

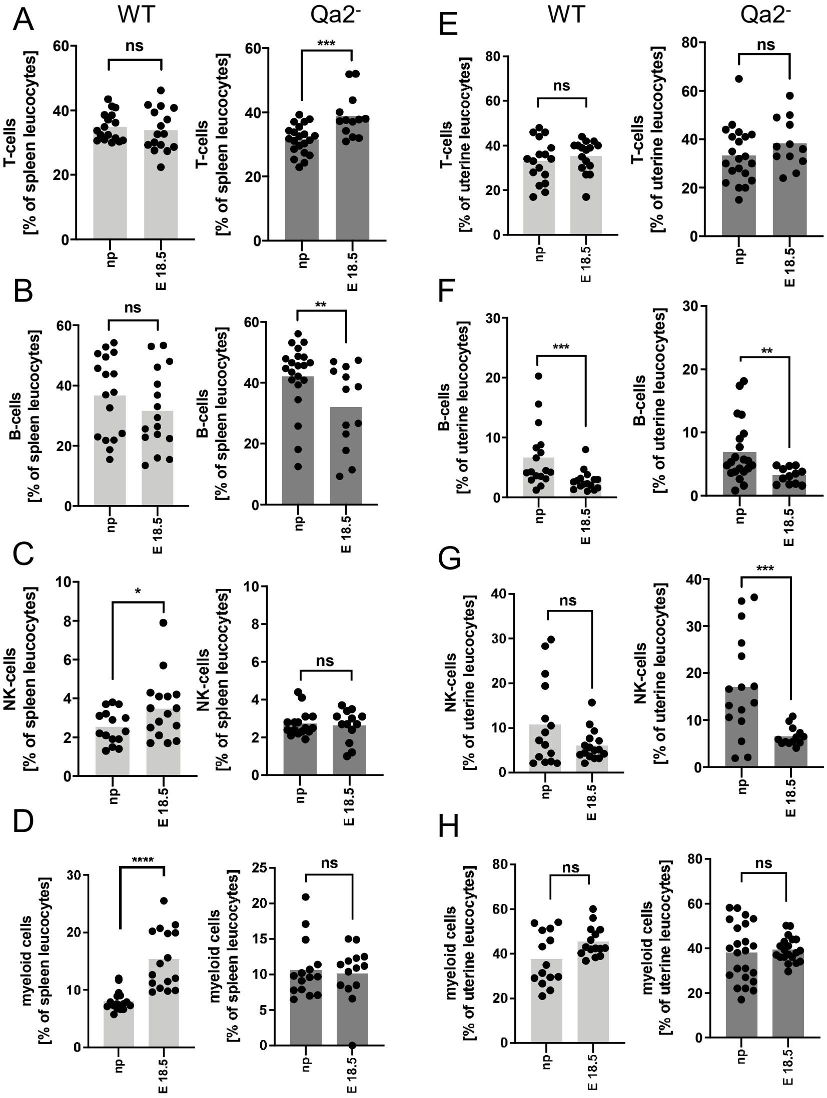

Supplementary Figure 5: Immune cell populations in spleens and uteri from wildtype and Qa-2 deficient mice

Wildtype (WT) and Qa-2 deficient mice (Qa2$^-$) were term-bred and the day when a vaginal plug was detected was defined as day E0.5. Mice were euthanized at E18.5 and spleens and uteri were collected. Non-pregnant animals served as controls. Tissues were homogenized and filtered to obtain single cell suspensions and cells were analyzed by flow cytometry. (A-H) Percentages of T-cells (A+E), B-cells (B+F) and NK-cells (C+G) and myeloid cells (D+H) from all spleen (A-D) and uterine (E-H) leucocytes in non-pregnant animals (np, n=14-17 for WT and n=16-21 for Qa-2$^-$) and pregnant animals at E18.5 (n=16 for WT and n=12-13 for Qa-2$^-$). Light grey graphs represent WT animals and dark grey graphs represent Qa-2$^-$ animals. Each symbol represents an individual animal and the mean is indicated. ****$p<0.0001$; ***$p<0.001$; *$p<0.05$; ns = not significant. Mann-Whitney test.

Supplementary Figure 6

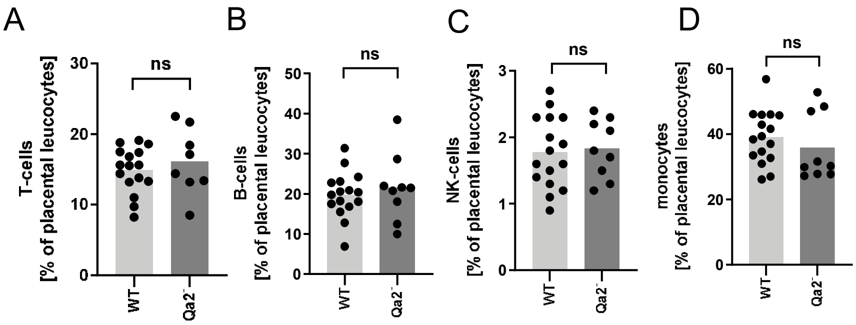

Supplementary Figure 6: Immune cell populations in placentas from wildtype and Qa-2 deficient mice

Wildtype (WT) and Qa-2 deficient mice (Qa2^{-}) were term-bred and the day when a vaginal plug was detected was defined as day E0.5. Mice were euthanized at E18.5 and placentas were collected. Tissues were homogenized and filtered to obtain single cell suspensions and cells were analyzed by flow cytometry. (A-E) Percentages of T-cells (A), B-cells (B) and NK-cells (C) and myeloid cells (D) of placenta leucocytes at E18.5 (n=16 for WT and n=8-9 for Qa-2^{-}). Light grey graphs represent WT animals and dark grey graphs represent Qa-2^{-} animals. Each symbol represents an individual animal and the mean is indicated. ns = not significant. Mann-Whitney test.

Supplementary Figure 7

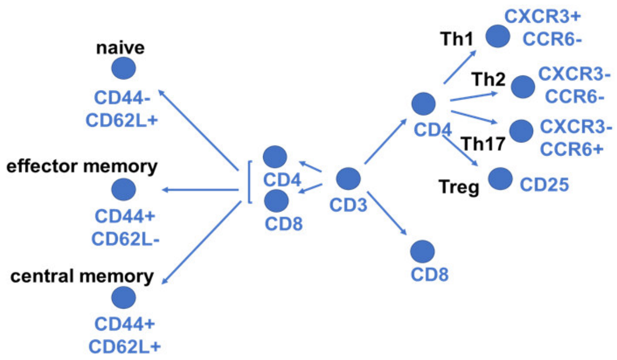

Supplementary Figure 7: Phenotyping strategy of T-cell subpopulations
